# The effects of COVID‐19 on child mental health: Biannual assessments up to April 2022 in a clinical and two general population samples

**DOI:** 10.1002/jcv2.12150

**Published:** 2023-03-30

**Authors:** Josjan Zijlmans, Jacintha M. Tieskens, Hedy A. van Oers, Hekmat Alrouh, Michiel A. J. Luijten, Rowdy de Groot, Daniël van der Doelen, Helen Klip, Rikkert M. van der Lans, Ronald de Meyer, Malindi van der Mheen, I. Hyun Ruisch, Germie van den Berg, Hilgo Bruining, Jan Buitelaar, Rachel van der Rijken, Pieter J. Hoekstra, Marloes Kleinjan, Ramón J. L. Lindauer, Kim J. Oostrom, Wouter Staal, Robert Vermeiren, Ronald Cornet, Lotte Haverman, Arne Popma, Meike Bartels, Tinca J. C. Polderman

**Affiliations:** ^1^ Department of Child and Adolescent Psychiatry & Psychosocial Care Amsterdam University Medical Center Vrije Universiteit Amsterdam Amsterdam The Netherlands; ^2^ Amsterdam Public Health Amsterdam University Medical Center Mental Health Amsterdam The Netherlands; ^3^ LUMC Curium – Child and Adolescent Psychiatry Leiden University Medical Center Leiden The Netherlands; ^4^ Child and Adolescent Psychiatry & Psychosocial Care Amsterdam University Medical Center University of Amsterdam Emma Children's Hospital Amsterdam The Netherlands; ^5^ Department of Biological Psychology Vrije Universiteit Amsterdam Amsterdam The Netherlands; ^6^ Epidemiology and Data Science Amsterdam University Medical Center Vrije Universiteit Amsterdam Amsterdam The Netherlands; ^7^ Department of Medical Informatics Amsterdam University Medical Center University of Amsterdam Amsterdam The Netherlands; ^8^ Karakter Child and Adolescent Psychiatry University Centre Nijmegen The Netherlands; ^9^ Praktikon Nijmegen The Netherlands; ^10^ Department of Child and Adolescent Psychiatry Amsterdam University Medical Center University of Amsterdam Amsterdam The Netherlands; ^11^ Levvel Academic Center for Child and Adolescent Psychiatry Amsterdam The Netherlands; ^12^ Department of Child and Adolescent Psychiatry University Medical Center Groningen, University of Groningen Groningen The Netherlands; ^13^ Netherlands Youth Institute Utrecht The Netherlands; ^14^ Department of Cognitive Neuroscience Donders Institute for Brain, Cognition and Behaviour Radboudumc Nijmegen The Netherlands; ^15^ Trimbos Institute Utrecht The Netherlands; ^16^ Utrecht University Interdisciplinary Social Sciences Youth Studies Utrecht The Netherlands; ^17^ Youz Parnassia Psychiatric Institute The Hague The Netherlands

**Keywords:** child mental health, COVID‐19, general population, pandemic, psychiatric care

## Abstract

**Background:**

The COVID‐19 pandemic has had an acute impact on child mental and social health, but long‐term effects are still unclear. We examined how child mental health has developed since the start of the COVID‐19 pandemic up to 2 years into the pandemic (April 2022).

**Methods:**

We included children (age 8–18) from two general population samples (*N* = 222–1333 per measurement and *N* = 2401–13,362 for pre‐covid data) and one clinical sample receiving psychiatric care (*N* = 334–748). Behavioral questionnaire data were assessed five times from April 2020 till April 2022 and pre‐pandemic data were available for both general population samples. We collected parent‐reported data on internalizing and externalizing problems with the Brief Problem Monitor and self‐reported data on Anxiety, Depressive symptoms, Sleep‐related impairments, Anger, Global health, and Peer relations with the Patient‐Reported Outcomes Measurement Information System (PROMIS®).

**Results:**

In all samples, parents reported overall increased internalizing problems, but no increases in externalizing problems, in their children. Children from the general population self‐reported increased mental health problems from before to during the pandemic on all six PROMIS domains, with generally worst scores in April 2021, and scores improving toward April 2022 but not to pre‐pandemic norms. Children from the clinical sample reported increased mental health problems throughout the pandemic, with generally worst scores in April 2021 or April 2022 and no improvement. We found evidence of minor age effects and no sex effects.

**Conclusions:**

Child mental health in the general population has deteriorated during the first phase of the COVID‐19 pandemic, has improved since April 2021, but has not yet returned to pre‐pandemic levels. Children in psychiatric care show worsening of mental health problems during the pandemic, which has not improved since. Changes in child mental health should be monitored comprehensively to inform health care and policy.

## Graphical abstract

We examined how child mental health has developed since the start of the pandemic up to 2 years into the pandemic. We found that child mental health in the general population deteriorated during the first phase of the pandemic, improved since April 2021, but has not yet returned to pre‐pandemic levels. Children in psychiatric care show worsening of mental health problems during the pandemic, which has not improved since.

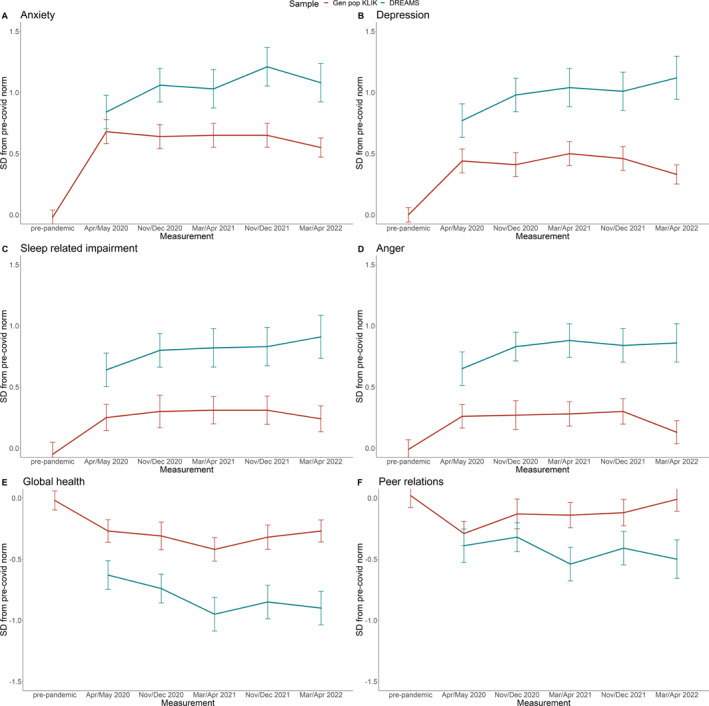

**Key points**

- Children from the general population reported increased mental health problems during the first year of the COVID‐19 pandemic. This has since improved, but not to pre‐pandemic levels.
- Children in psychiatric care reported increased mental health problems throughout the pandemic, which have not improved up to April 2022.
- The pandemic did not impact the mental health of boys and girls in our samples differently.
- As there is large variability in how children reacted to the pandemic, future studies should study determinants of better and worse outcomes.
- Further changes in child mental health should be monitored comprehensively to aid health care and policy.

## INTRODUCTION

The COVID‐19 pandemic and subsequent restrictions such as social distancing, the closing of schools, and even total lockdowns severely disrupted people's lives. Children and adolescents (hereafter referred to as children) are less susceptible to physical symptoms of COVID‐19 infections (Viner et al., 2021). However, they may be more prone to negative indirect effects of the pandemic such as lockdowns and other restrictions that could disrupt their social networks and may be at risk of developing mental health problems (Danese et al., 2020).

Research into the effects of disasters and emergencies, such as pandemics, wars, and natural disasters, has shown that children's resilience to traumatic events varies greatly. For instance, increases in mental health problems in response to traumatic experiences rapidly decrease to normal levels in some children, while for other children the consequences can be long‐lasting (Masten & Motti‐Stefanidi, 2020; Sonuga‐Barke & Fearon, 2021). During the pandemic, children with pre‐existing mental health problems may be particularly at risk of sustained negative mental health effects, as their pre‐existing mental health problems might exacerbate and as their mental healthcare was often interrupted or altered (Hoffmann & Duffy, 2021; Witt et al., 2020). Evidence for an increase in mental problems in such vulnerable groups was reported in a 12‐month longitudinal study in over 34,000 UK children in the first year of the pandemic (Parsons et al., 2022). In addition, some studies have reported that girls and older children (13–15 compared to 6–12 years old) may be more strongly impacted by the pandemic (Panchal et al., 2021), but as pre‐pandemic data are often missing, it is hard to conclude whether these are COVID‐specific or general effects.

Indeed, systematic reviews and meta‐analyses conclude that the mental health of children worldwide was negatively affected during the first year of the COVID‐19 pandemic (Ma et al., 2021; Panchal et al., 2021; Samji et al., 2022; Zolopa et al., 2022), but these interpretations were mostly based upon cross‐sectional studies performed during the pandemic. Prospective studies on child mental health during the pandemic that include pre‐pandemic measurements are scarce but do seem to confirm that predominantly affective problems such as depressive, anxious, and stress symptoms worsened in the first months after the onset of the COVID‐19 pandemic. This seems to be the case for children from the general population (Barendse et al., 2022; Bignardi et al., 2021; de France et al., 2022; Fischer et al., 2022; Luijten, van Muilekom, et al., 2021; Ravens‐Sieberer et al., 2022; van der Velden et al., 2022; von Soest et al., 2022) as well as for children with pre‐existing mental health problems (Breaux et al., 2021; Fischer et al., 2022; Gabriel et al., 2022). On the contrary, some other studies challenge the mental burden of the COVID‐19 pandemic in children and report small or no significant differences in mental health problems (Bouter et al., 2022; Burdzovic Andreas & Brunborg, 2021), which might be explained by the relatively short time frame of both studies (respectively from April 2020 to January 2021, and April 2020 to July 2020).

Thus far, little data exists on the effects of the pandemic on child mental health past early 2021 (the first year of the pandemic). However, to be able to provide adequate measures and counseling, it is important for policy makers and health care professionals to know whether problems have exacerbated, stabilized, or improved since then. Therefore, we aimed to (1) quantify how child mental health has developed since the start of the COVID‐19 pandemic up to 2 years into the pandemic (April 2022) in children from the general population and in children with pre‐existing problems receiving psychiatric care; and (2) investigate whether COVID‐related mental health changes are moderated by sex and age. We systematically examined the mental health of children from the general population and children in psychiatric care using existing data from before the COVID‐19 pandemic and gathered new data over the course of the pandemic biannually from April 2020 till April 2022. We collected parent‐reported and self‐reported outcome measures on multiple domains of mental health, including internalizing and externalizing problems.

## METHODS

### Participants

The Dutch consortium Child and Adolescent Mental Health and WellBeing in times of the COVID‐19 pandemic (CAMHWB‐19) was initiated to assess the impact of the COVID‐19 pandemic on the mental health and wellbeing of children and adolescent in the Netherlands. It comprises (parents of) children aged 8–18 from two general population samples and one clinical sample. We previously reported on the first two pandemic measurements of this study (Fischer et al., 2022). The two population samples are (1) The Netherlands Twin Register (NTR; Ligthart et al., 2019), a twin cohort that has collected data over the past 35 years in the general Dutch population (age 8–17 in the current study); (2) The KLIK group, which aimed to collect samples representative of the Dutch population using an online panel agency (age 8–18). The clinical sample is (3) DREAMS (Dutch Research in child and Adolescent Mental health), a collaboration between four academic child and adolescent psychiatry centers (Amsterdam, Groningen, Leiden, Nijmegen) in the Netherlands (age 8–18). DREAMS has obtained information particularly for this study from children and their parents receiving psychiatric care for varying problems, including autism, ADHD, and depression. Sample sizes varied between 222 and 1333 for each measurement during the COVID pandemic and are up to 13,362 before the COVID pandemic.

Collaborating parties received approval for data collection by the appropriate ethics committees and all children and parents provided informed consent. The studies were conducted in line with the ethical standards stated in the 1964 Declaration of Helsinki and its later amendments.

### Procedure

Data were collected at five moments in time after the start of the pandemic, approximately once every 6 months. At each moment, both new and recurrent participants were invited to participate. To prevent within‐subject effects biasing the results, in all samples we randomly selected one measurement occasion for each individual participant, realizing a repeated cross‐sectional design. Pre‐pandemic data were available for the two general population samples, but not for the clinical sample. Table 1 presents an overview of the samples and data that were used for the analyses.

**TABLE 1:** Sociodemographic characteristics of the samples.

Cohort	0	1	2	3	4	5
	pre‐pandemic	Apr/May 2020	Nov/Dec 2020	Mar/Apr 2021	Nov/Dec 2021	Mar/Apr 2022
NTR						
*N* total	13,950	1791	536	714	855	883
*N* after random selection^a^	13,362	1333	222	347	426	458
Male	49.5%	49.3%	49.6%	55.0%	47.4%	49.6%
Female	50.5%	50.7%	50.4%	45.0%	52.6%	50.4%
*M* Age (SD)	11.6 (1.3)	10.7 (1.8)	10.3 (2.0)	10.1 (2.1)	10.9 (2.2)	11.1 (1.9)
Country of birth parents (both Dutch)^b^	88.8%	83.4%	83.3%	73.8%	83.8%	81.2%
Educational level parents low^c^	20.5%	3.9%	1.4%	5.2%	0.0%	4.8%
Educational level parents intermediate^c^	38.2%	29.9%	38.6%	31.0%	30.0%	14.3%
Educational level parents high^c^	41.3%	66.2%	60.0%	63.8%	70.0%	81.0%
KLIK						
*N* total	2401	856	939	909	828	893
*N* after random selection^a^	2401	486	440	413	414	529
Male	50.3%	47.9%	50.2%	52.1%	50.5%	54.1%
Female	49.7%	52.1%	49.8%	47.9%	49.5%	45.9%
*M* Age (SD)	13.1 (3.1)	13.5 (2.9)	13.8 (3.2)	13.6 (3.3)	13.6 (3.1)	13.4 (3.1)
Country of birth parents (both Dutch)	93.0%^d^	92.6%	92.0%	88.6%	94.7%	90.7%
Educational level parents low	19.3%^d^	2.9%	0.6%	4.2%	1.9%	1.2%
Educational level parents intermediate	39.0%	28.2%	29.4%	26.6%	25.2%	22.3%
Educational level parents high	41.6%	68.8%	70.0%	69.2%	72.9%	76.5%
DREAMS						
*N* total	–	500	892	661	632	450
*N* after random selection^a^	–	453	748	505	482	334
Male	–	54.3%	53.5%	59.6%	53.7%	47.0%
Female	–	45.7%	46.5%	40.4%	46.3%	53.0%
*M* Age (SD)	–	13.3 (3.1)	13.6 (2.9)	13.0 (2.9)	13.2 (3.0)	13.3 (2.9)
Country of birth parents (both Dutch)	–	84.3%	88.4%	86.2%	85.4%	84.5%
Educational level parents low	–	4.8%	5.6%	6.3%	6.9%	6.5%
Educational level parents intermediate	–	40.3%	45.6%	42.4%	44.4%	38.1%
Educational level parents high	–	54.9%	48.8%	51.3%	48.7%	55.4%

*Note*: Statistics represent the samples *after* random selection of a single measurement moment for each participant.

Abbreviations: M, mean; N, number of participants; SD, standard deviation.

^a^ Number of participants after random selection of one measurement for each individual participant.

^b^ 4%–13% of data are missing per measurement. Shown percentages are valid percentages (excluding missing cases).

^c^ 4%–25% of data are missing per measurement. Shown percentages are valid percentages (excluding missing cases).

^d^ Sample size is smaller (*N* = 1082) as data were not collected in norm studies for PROMIS anxiety and depressive symptoms.

**FIGURE 1:**
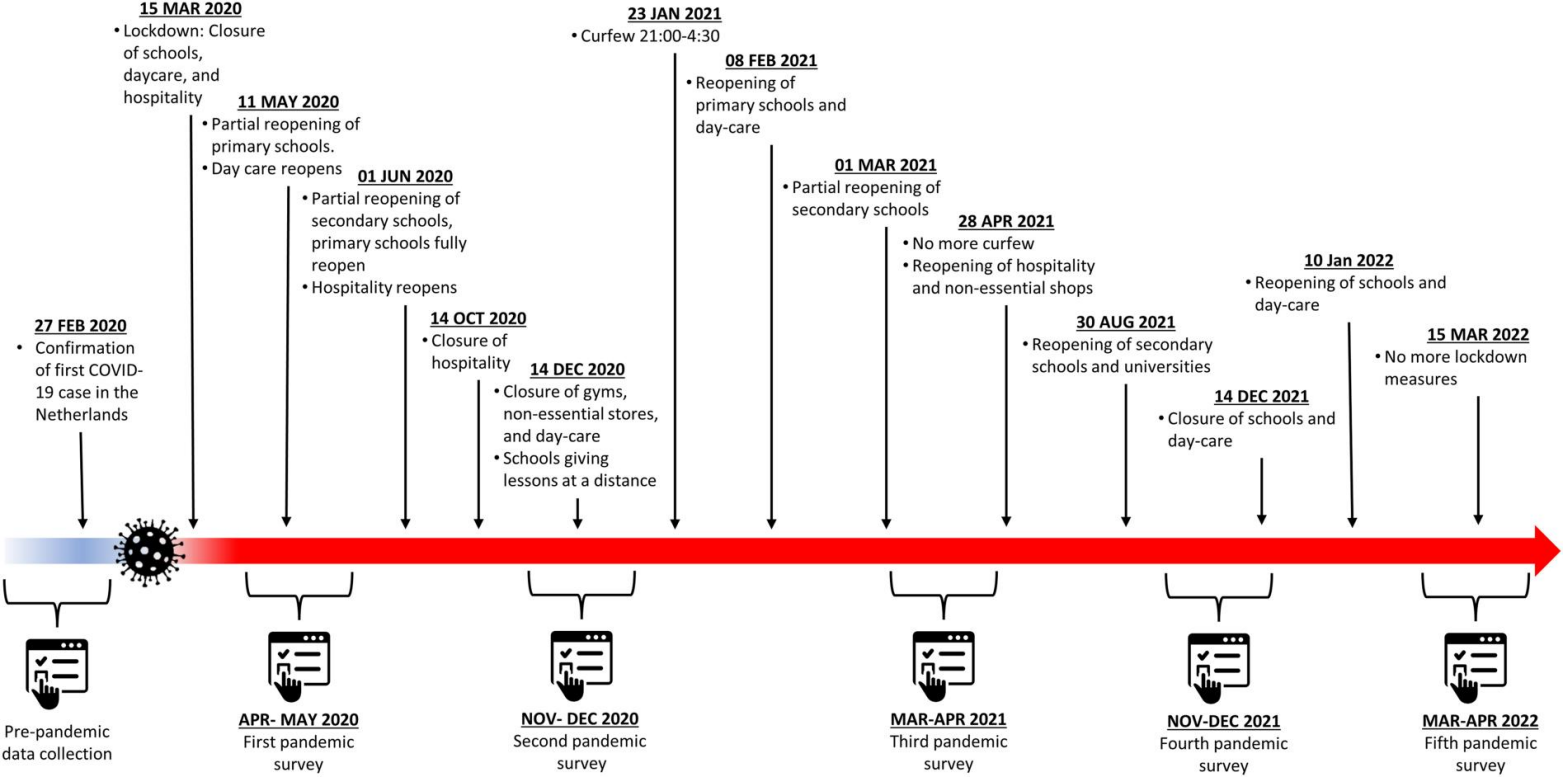
Timeline of COVID‐19 regulations in the Netherlands.

Data were collected in April/May 2020 (measurement 1), November/December 2020 (measurement 2), March/April 2021 (measurement 3), November/December 2021 (measurement 4), and March/April 2022 (measurement 5). Measurement 1 was during the first peak of the pandemic when the first lockdown was set in The Netherlands and all schools were closed. Measurement 2 was during a partial lockdown with schools partially reopened. Measurement 3 was also during partial lockdown with the addition of a nighttime curfew. Measurement 4 was again during (partial) lockdown, schools were still open but right after our data collection schools closed again (on December 14). Measurement 5 was during a relaxation of most of the restrictions. Schools were open, people were allowed to work from the office, and facemasks were no longer mandatory in most public spaces. See Figure 1 for an overview of the data collection and Dutch COVID restrictions at the time.

NTR invited parents of twins via email for a regular survey (measurement 2) and for four COVID specific additional surveys. For analyses, one child of a twin pair was always randomly selected to be included to avoid dependencies. Pre‐pandemic data were available from previous standard cohort data collections from 1998 until 2019. Samples drawn during the pandemic are similar but include more children from high‐education families (see Table 1). Response rates were chronologically 45%, 38%, 31%, 31%, 19%, 14%.

KLIK invited children and their parents via an independent, online survey panel (PanelInzicht) (Luijten, van Litsenburg, et al., 2021). Data were collected through a research website of the KLIK portal (www.corona.hetklikt.nu). Pre‐pandemic data collected between December 2017 and April 2018 were available from previous validation studies of the PROMIS measures. The pre‐pandemic group was representative of the Dutch general population within 2.5% on most key demographics (age, gender, ethnicity, region, and educational level) compared to population numbers in 2017. During sampling, when particular demographics were underrepresented, the panel agency invited more people from these demographics until they were properly represented. Samples drawn during the pandemic are similar but include slightly more children of whom both parents were born in the Netherlands and slightly fewer children from low‐education families (see Table 1).

DREAMS invited children receiving psychiatric care and their parents via e‐mail through their respective psychiatric centers. Data were collected via the same research portal as the KLIK group employed. Response rates were respectively 9%, 10%, 11%, 10%, and 9%.

## MEASURES

### Socio‐demographic information

To describe the samples, we gathered data on the country of birth and educational level of the parents. Country of birth was operationalized as both parents being born in the Netherlands (yes/no). Educational level was operationalized as the highest education among both parents (Low = primary, lower vocational education, lower and middle general secondary education; Intermediate = middle vocational education, higher secondary education, pre‐university education; High = higher vocational education, university).

### Parent‐reported outcomes

For parental reports in NTR and DREAMS, we employed the Brief Problem Monitor of the Achenbach System of Empirical Based Assessment (ASEBA‐BPM). The BPM (Achenbach et al., 2011) is a shortened version of the Child Behavior Checklist (CBCL/6–18 years (Achenbach & Rescorla L A, 2001). It assesses behavioral and emotional problems in children as reported by their parents. Items are rated on a three‐point Likert‐scale, where parents rate if a statement applies to their child (0 = ‘not true’, 1 = ‘somewhat true’, 2 = ‘very true’). In line with the BPM manual, we coded missing items on the BPM as zero. If more than 20% of items were missing, we excluded the participant from the BPM analysis. The BPM yields an internalizing score calculated from six items and an externalizing score. The externalizing score usually is calculated from seven items, but because one item pertains to behavior at school and data were also collected when children did not go to school, we excluded this item. The six remaining items were weighted so that the sum score has the same range as the normal scoring to allow comparison to other studies.

### Child‐reported outcomes

For child self‐reports in KLIK and DREAMS, we employed the *Patient‐Reported Outcomes Measurement Information System (PROMIS®)*. Six measures of the Dutch‐Flemish PROMIS® were used to assess self‐reported Anxiety v2.0 (Irwin et al., 2010), Depressive Symptoms v2.0 (Irwin et al., 2010), Anger v2.0 (Irwin et al., 2012), Sleep‐related impairment v1.0 (Forrest et al., 2018), Global health v1.0 (Forrest et al., 2014), and Peer Relationships v2.0 (DeWalt et al., 2013). All instruments except Anger and Global Health were administered as Computerized Adaptive Tests (CAT), where items are selected based on responses to previously completed items, resulting in reliable scores with fewer items (Cella et al., 2007). PROMIS measures use a 7‐day recall period, and most items are scored on a five‐point Likert scale ranging from ‘never’ to ‘(almost) always’. Total scores are calculated by transforming item scores into T‐scores ranging from 0 to 100 with a mean of 50 and standard deviation of 10 in the original calibration sample; a mixed sample of a general and a clinical population from the U.S (Irwin et al., 2010). The US item parameters were used in the CAT algorithm and T‐score calculations, as by PROMIS convention. The PROMIS pediatric item banks and scales have previously been validated in the Dutch population (Klaufus et al., 2021; Luijten et al., 2022; Luijten, van Litsenburg, et al., 2021; Peersmann et al., 2022; van Muilekom et al., 2021).

### Data analysis

Statistical analyses were performed in IBM SPSS Statistics 28. Within each sample and for each outcome variable, we performed analyses of covariance (ANCOVA) to test whether mental health problems were different over the course of the pandemic. Age, and sex were included as covariates, and we tested for interaction effects between time and sex and time and age. For the latter interaction, we split age into two groups: children below the age of 12 years old and of age 12 and higher. In addition, country of birth of the parents (both Dutch), educational level parents (low/medium/high) were added as covariates to the analyses within the DREAMS sample and within the KLIK sample when these were available (for all outcomes except anxiety and depression). In the NTR sample, due to relatively high missingness (up to 39% combined) on these variables we report uncorrected results in the main manuscript and performed a supplementary analysis that included country of birth and education to test whether this impacted results. We performed post‐hoc Least Significant Differences tests to compare individual measurement moments within each sample. For the BPM measures, we report differences in scores expressed as estimated marginal means (EMM) of Z‐scores standardized to pre‐pandemic data of the NTR for ease of interpretation (see Table 2). To facilitate comparison to other (international) studies, sum scores are presented in Table S1. Likewise, for the PROMIS measures, we report differences in scores expressed as EMMs of Z‐scores standardized to pre‐pandemic norm scores for KLIK in Table 2, and for international comparison, T‐scores based on the original (United States) calibration sample are reported in Table S2. As the main goal of this study is merely establishing a general overview of mental health in children and adolescents during the COVID pandemic, without testing specific hypotheses, we do not correct for multiple testing.

**TABLE 2:** BPM and PROMIS standardized estimated marginal means (EMM), standard errors, and comparisons between measurement points.

Cohort	0 (a)	1 (b)	2 (c)	3 (d)	4 (e)	5 (f)
	pre‐pandemic	Apr/May 2020	Nov/Dec 2020	Mar/Apr 2021	Nov/Dec 2021	Mar/Apr 2022
NTR						
*N*	13,341	1332	221	347	426	458
BPM internalizing	0.00 (0.01)^bcdef^	0.38 (0.03)^adef^	0.24 (0.07)^adef^	0.6 (0.06)^abc^	0.55 (0.05)^abc^	0.56 (0.05)^abc^
BPM externalizing	0.01 (0.01)	0.03 (0.03)	0.01 (0.07)	0.11 (0.06)	0.06 (0.05)	0.05 (0.05)
DREAMS						
*N*	–	404	599	445	413	295
BPM internalizing	–	2.76 (0.14)^def^	2.88 (0.13)^de^	3.21 (0.14)^bc^	3.21 (0.14)^bc^	3.2 (0.15)^c^
BPM externalizing	–	1.36 (0.1)	1.46 (0.09)	1.55 (0.1)	1.49 (0.1)	1.52 (0.11)
KLIK						
*N*	527–1082^*^	471–486	425–440	407–413	401–414	514–529
Anxiety^†^ ^,^ ^§^	−0.02 (0.03)^bcdef^	0.68 (0.05)^af^	0.64 (0.05)^a^	0.65 (0.05)^a^	0.65 (0.05)^a^	0.55 (0.04)^ab^
Depressive symptoms^†^ ^,^ ^§^	0.00 (0.03)^bcdef^	0.44 (0.05)^a^	0.41 (0.05)^a^	0.50 (0.05)^af^	0.46 (0.05)^af^	0.33 (0.04)^ade^
Sleep‐related impairments^†^	−0.05 (0.05)^bcdef^	0.25 (0.06)^a^	0.30 (0.07)^a^	0.31 (0.06)^a^	0.31 (0.06)^a^	0.24 (0.05)^a^
Anger^†^	−0.01 (0.04)^bcdef^	0.26 (0.05)^af^	0.27 (0.06)^af^	0.28 (0.05)^af^	0.30 (0.05)^af^	0.13 (0.05)^abcde^
Global health^‡^	−0.02 (0.04)^de^	−0.27 (0.05)^d^	−0.31 (0.06)	−0.42 (0.05)^abf^	−0.32 (0.05)^a^	−0.27 (0.05)^d^
Peer relations^‡^	0.05 (0.04)^bcde^	−0.29 (0.05)^acdef^	−0.13 (0.06)^ab^	−0.14 (0.05)^abf^	−0.12 (0.06)^ab^	−0.01 (0.05)^bd^
DREAMS						
*N*	–	241–257	434–456	236–250	199–216	169–181
Anxiety^†^	–	0.84 (0.07)^cdef^	1.06 (0.07)^b^	1.03 (0.08)^be^	1.21 (0.08)^bd^	1.08 (0.08)^b^
Depressive symptoms^†^	–	0.77 (0.07)^cdef^	0.98 (0.07)^b^	1.04 (0.08)^b^	1.01 (0.08)^b^	1.12 (0.09)^b^
Sleep‐related impairments^†^	–	0.64 (0.07)^cdef^	0.80 (0.07)^b^	0.82 (0.08)^b^	0.83 (0.08)^b^	0.91 (0.09)^b^
Anger^†^	–	0.65 (0.07)^cdef^	0.83 (0.06)^b^	0.88 (0.07)^b^	0.84 (0.07)^b^	0.86 (0.08)^b^
Global health^‡^	–	−0.63 (0.06)^def^	−0.74 (0.06)^d^	−0.95 (0.07)^bc^	−0.85 (0.07)^b^	−0.90 (0.07)^b^
Peer relations^‡^	–	−0.39 (0.07)	−0.32 (0.06)^de^	−0.54 (0.07)^c^	−0.41 (0.07)	−0.50 (0.08)^c^

*Note*: ^a,b,c,d,e,f^ represent significant differences at *p* < 0.05 between measurements using Least Significant Differences post‐hoc tests. For example, superscript ^b^ in column (d) indicates a significant post‐hoc difference between columns (b) and (d) for a variable.

^*^ Sample sizes vary because data from different domains comes from different norm studies.

^†^ Higher scores indicate more symptoms.

^‡^ Higher scores indicate better functioning.

^§^ Analyses are not controlled for birth country of parents and educational level parents due to missing data.

Finally, we report the proportions of children who scored outside of the normal range on the BPM internalizing and externalizing scales based on rater and sex specific T‐scores (*T* > 65) in Table S1 and the proportion of children who scored outside of the normal range on the PROMIS scales in Table S3.

## RESULTS

### Parent‐reported outcomes (Brief Problem Monitor)

**FIGURE 2:**
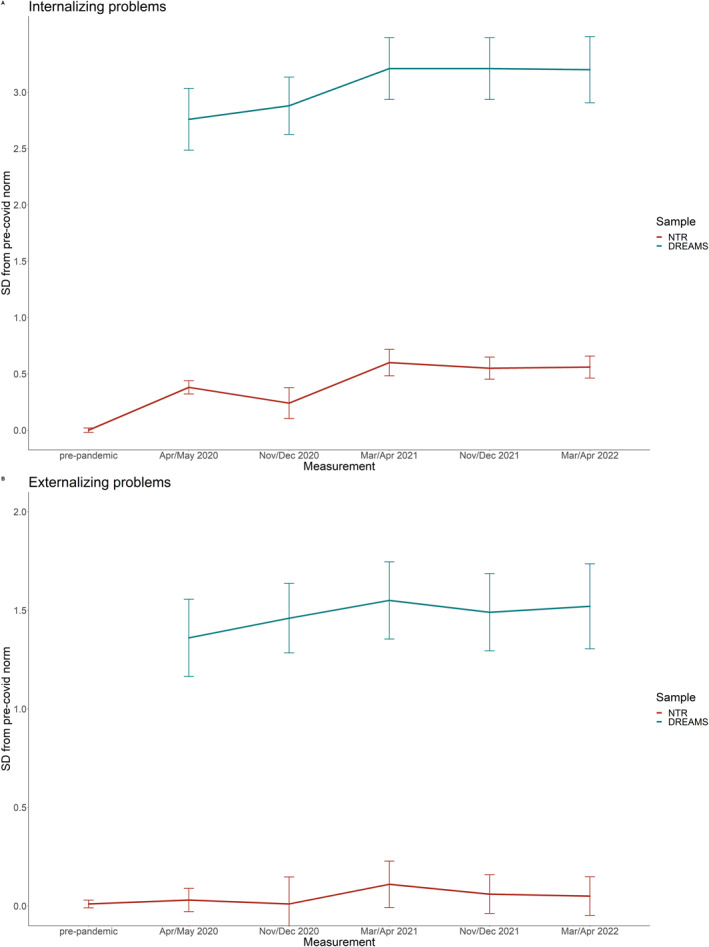
Estimated marginal means of parent‐reported outcomes (BPM).

Table 2 presents results for the BPM outcome measures of NTR and DREAMS, and Figure 2 illustrates the EMMs of the general population sample and clinical sample over time, represented as standard deviations from pre‐covid NTR norm scores.

In the general population sample of NTR, internalizing problems differed significantly between measurements (*F* = 80.61, *p* < .001), with lowest scores during the pre‐COVID measurement and highest scores during the third COVID measurement (March/April 2021). The final COVID measurement (March/April 2022) had significantly higher scores than the pre‐pandemic measurement and did not differ from the measurement with the highest scores. There was a significant interaction between time and age (*F* = 4.25, *p* < .001), where younger children had a steeper increase in problems during the first COVID measurement (April/May 2020) and then returned to the same pattern as the older children. There was no interaction between time and sex.

Externalizing problems did not significantly differ between measurements. There was a significant interaction between time and age (*F* = 3.28, *p* < .01), where younger children varied little between measurements and older children more so, but both groups did not change from pre‐covid to the final measurement. There were no interaction effects between time and sex.

Supplementary analyses that included pre‐pandemic data only up to 5 years before the pandemic showed a highly similar pattern of results for internalizing problems. These differed significantly between measurements (*F* = 30.31, *p* < .001) and there was a significant interaction between time and age (*F* = 3.01, *p* < .01). The results for externalizing problems showed a different pattern, where externalizing problems differed significantly over time (*F* = 5.67, *p* < .001) with lowest scores during the pre‐COVID measurement and highest scores during the third COVID measurement (March/April 2021). The final COVID measurement (March/April 2022) had significantly higher scores than the pre‐pandemic measurement and did not differ from the measurement with the highest scores. See Table S4 for the detailed findings.

Supplementary analyses that included parental country of birth and parental education showed an identical pattern of results. Internalizing problems differed significantly between measurements (*F* = 53.74, *p* < .001) and there was a significant interaction between time and age (*F* = 2.395, *p* < .05). Externalizing problems did not significantly differ between measurements and there was a significant interaction between time and age (*F* = 2.685, *p* < .05).

In the clinical sample of DREAMS (note that there were no pre‐pandemic data available for this sample), internalizing problems differed significantly between measurements (*F* = 3.76, *p* < 0.01), with lowest problem scores during the first COVID measurement (April/May 2020) and highest scores during the third COVID measurement (March/April 2021). The final COVID measurement (March/April 2022) had significantly higher scores than the first COVID measurement and did not differ from the measurement with the highest scores. There were no interaction effects between time and age or time and sex.

Externalizing problems did not significantly differ between measurements. There were no interaction effects between time and age or time and sex.

### Child‐reported outcomes (PROMIS)

**FIGURE 3:**
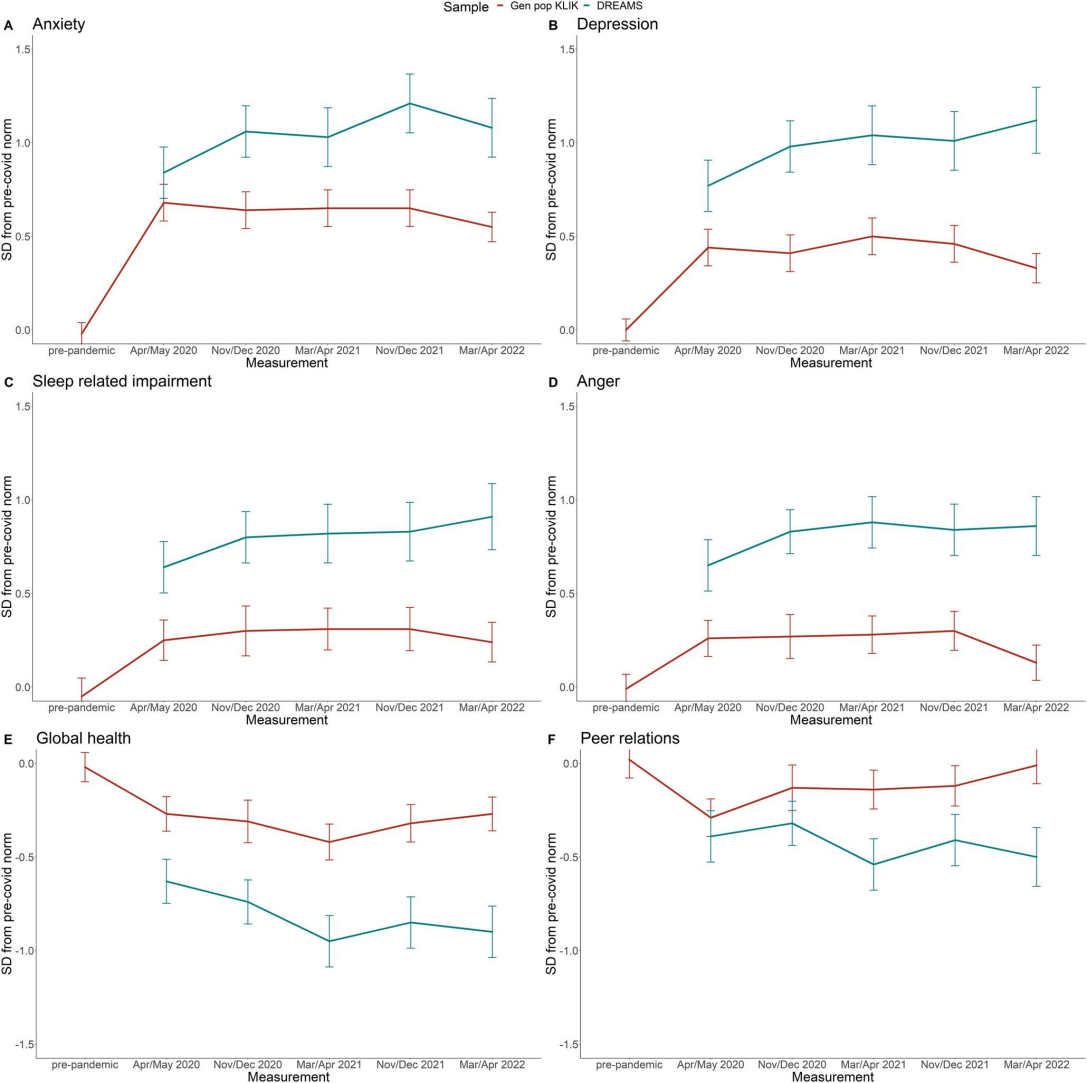
Estimated marginal means of child‐reported outcomes (PROMIS).

Table 2 presents results for the PROMIS outcome measures of the KLIK and DREAMS samples, and Figure 3 illustrates the EMMs of the general population sample and clinical sample over time, represented as standard deviations from pre‐covid norm scores. Table S2 shows the EMMs of the U.S. calibrated T‐scores for international comparison.

In the KLIK sample, levels of anxiety differed significantly between measurements (*F* = 74.69, *p* < .001), with lowest problem scores during the pre‐pandemic measurement and highest scores during the first COVID measurement (April/May 2020). The final COVID measurement (March/April 2022) had significantly higher scores than the pre‐pandemic measurement and significantly lower scores than the measurement with the highest scores.

Levels of depression differed significantly between measurements (*F* = 33.13, *p* < .001), with lowest scores during the pre‐pandemic measurement and highest scores during the third COVID measurement (March/April 2021). The final COVID measurement (March/April 2022) had significantly higher scores than the pre‐pandemic measurement and significantly lower scores than the measurement with the highest scores.

Sleep‐related impairments differed significantly between measurements (*F* = 4.66, *p* < .001), with lowest scores during the pre‐pandemic measurement and highest scores during the third COVID measurement (March/April 2021). The final COVID measurement (March/April 2022) had significantly higher scores than the pre‐pandemic measurement and did not differ from the measurement with the highest scores. There was a significant interaction between time and age (*F* = 2.98, *p* < .01), where younger children showed a larger increase in impairment from the pre‐pandemic measurement to the first COVID measurement (April/May 2020).

Anger differed significantly between measurements (*F* = 6.05, *p* < .001), with lowest scores during the pre‐pandemic measurement and highest scores during the third COVID measurement (March/April 2021). The final COVID measurement (March/April 2022) had significantly higher scores than the pre‐pandemic measurement and significantly lower scores than the measurement with the highest scores.

Global health differed significantly between measurements (*F* = 2.34, *p* < .05), with best scores during the pre‐pandemic measurement and worst scores during the third COVID measurement (March/April 2021). The final COVID measurement (March/April 2022) did not differ from the pre‐pandemic measurement and had significantly better scores than the measurement with the worst scores. There was a significant interaction between time and age (*F* = 2.42, *p* < .05), where older children improved after the third COVID measurement (March/April 2021), but younger children did not.

Peer relations differed significantly between measurements (*F* = 4.67, *p* < .001), with best scores during the pre‐pandemic measurement and worst scores during the first COVID measurement (April/May 2020). The final COVID measurement (March/April 2022) did not differ from the pre‐pandemic measurement and had significantly better scores than the measurement with the worst scores. There was a significant interaction between time and age (*F* = 2.31, *p* < .05) where younger children showed a larger decrease in peer relations from the pre‐pandemic measurement to the first COVID measurement (April/May 2020).

Apart from the interactions for sleep‐related impairments and global health, none of the interactions between time and age and time and sex on PROMIS outcomes were significant.

In the DREAMS sample (note that there were no pre‐pandemic data available for this sample), levels of anxiety differed significantly between measurements (*F* = 4.74, *p* < .001), with lowest scores during the first COVID measurement (April/May 2020) and highest scores during the fourth COVID measurement (November/December 2021). The final COVID measurement (March/April 2022) had significantly higher scores than the first COVID measurement and did not differ from the measurement with the highest scores.

Levels of depression differed significantly between measurements (*F* = 4.14, *p* < .01), with lowest scores during the first COVID measurement (April/May 2020) and highest scores during the final COVID measurement (March/April 2022). The final COVID measurement had significantly higher scores than the first COVID measurement.

Sleep‐related impairments differed significantly between measurements (*F* = 2.31, *p* < .05), with lowest scores during the first COVID measurement (April/May 2020) and highest scores during the final COVID measurement (March/April 2022). The final COVID measurement had significantly higher scores than the first COVID measurement.

Anger differed significantly between measurements (*F* = 2.84, *p* < .05), with lowest scores during the first COVID measurement (April/May 2020) and highest scores during the third COVID measurement (March/April 2021). The final COVID measurement (March/April 2022) had significantly higher scores than the first COVID measurement and did not differ from the measurement with the highest scores.

Global health differed significantly between measurements (*F* = 5.72, *p* < .001), with best scores during the first COVID measurement (April/May 2020) and worst scores during the third COVID measurement (March/April 2021). The final COVID measurement (March/April 2022) had significantly worse scores than the first COVID measurement and did not differ from the measurement with the worst scores.

Peer relations differed significantly between measurements (*F* = 2.49, *p* < .05), with best scores during the second COVID measurement (November/December 2020) and worst scores during the third COVID measurement (March/April 2021). The final COVID measurement (Mar/Apr 2022) did not differ from the first COVID measurement and had significantly worse scores than the measurement with the best scores.

None of the interactions between time and age and time and sex were significant.

## DISCUSSION

In this study we assessed parent‐reported and self‐reported child mental and social health at five cross‐sectional measurements during the pandemic in two samples of the general population and one clinical sample in the Netherlands (age 8 to 18).

### General population samples

In the NTR sample, for which we have parent‐reported data, no differences were observed in externalizing problems from pre‐pandemic to pandemic measurements nor over the course of the pandemic. However, parents did report higher internalizing problems of their children during the pandemic compared to before the pandemic, and in April 2022 this had not improved. In the KLIK sample for which we have self‐reported data, we observed significant increases in mental health problems from before the pandemic to the first measurement during the pandemic (April 2020) on all six mental health domains. In line with previous research, we found the largest increases in anxiety and depression (Barendse et al., 2022; Bignardi et al., 2021, de France et al., 2022). After this initial deterioration the problems stabilized and, in most domains, started to improve toward the final measurement. All measures except for sleep‐related impairment were significantly better in April 2022 compared to the worst measurement during COVID (either April 2020 or April 2021). However, on all domains, except for peer relations, children still reported significantly more problems in April 2022 than before the pandemic. This differs from the parent‐report of the NTR, where no improvement was visible after the initial decline. Similar patterns were found in a recent German study in over 1500 families where children aged 11–17 self‐reported increasing symptoms of anxiety and depression up to December 2020 which decreased toward October 2021 but not to pre‐pandemic levels. However, parents in the same study reported in their children aged 7–17 increases in emotional problems, conduct problems, hyperactivity, and peer problems that had not improved toward October 2021 (Ravens‐Sieberer et al., 2022). In another large‐scale study from Norway (*N* = 227,258), adolescents aged 13–18 reported slightly higher depressive symptoms up to March 2021 and no differences in conduct problems and satisfaction with social relationships (von Soest et al., 2022).

### Clinical sample

In the clinical sample, we found self‐reported mental health to worsen throughout the pandemic on all mental health domains except peer relations, which remained stable. Like the self‐reports of the general population, differences were largest for anxiety and depression. Contrary to the general population, by April 2022 none of the measures had significantly improved compared to the worst measurement during COVID and on all mental health domains (except peer relations) children reported significantly more problems than during the first COVID measurement in April 2020. The parent‐reported outcomes regarding internalizing problems are similar, although the effects seem delayed; parents reported increased internalizing problems from April 2021 onwards, but not before. In previous work we discussed that internalizing problems may be less readily noticed by parents or may be perceived as less problematic (Fischer et al., 2022). However, the current data suggest that parents do notice internalizing problems of their children, although in a later stage of the pandemic than children themselves. Like in the general population, parents of children from the clinical sample did not report differences in externalizing problems throughout the pandemic. Our results indicate that children in psychiatric care recuperate more slowly from the effects of the pandemic than children from the general population. Possibly, this is because children in psychiatric care are a vulnerable population that may be less resilient to substantial stressors like the COVID‐pandemic and may find it harder to adjust to society and lockdown measures changing at a high frequency (Sonuga‐Barke & Fearon, 2021). Another factor may be that although pandemic measures have been lifted, it takes time to alleviate the effects of disrupted care (Hoffmann & Duffy, 2021). Mental health care systems themselves are still recuperating and are now treating children with increased problems compared to before the pandemic. Likely, this has resulted in a higher burden for mental health care.

### Sex and age effects

Whereas other studies have suggested that girls and older children may be more vulnerable to effects of the pandemic (Panchal et al., 2021), we did not find evidence that problems increased more in girls or adolescents throughout the pandemic. As most published studies reported on single cross‐sectional measurements, we suspect many reported effects of age and sex to be non‐specific to the COVID‐19 pandemic. Nonetheless, some longitudinal studies have observed more pronounced changes in girls. For example, Magson et al. (2021) reported greater increases in depressive and anxious symptoms in adolescent girls compared to adolescent boys (age 13–16). It is possible that our broader age range makes it harder to detect sex effects, and it is a limitation of our study that we lacked statistical power to look at changes in mental health in specific sex and age groups. We suggest that more focused studies are needed to reveal these. We did find minor differences between age groups in mental health progression throughout the pandemic, where younger children showed faster increases in sleep‐related problems and slower recuperation of global health. However, the effects are small, and no consistent pattern emerges from the different measures. As there is much variability in how children have reacted to the pandemic (also see Parsons et al., 2022), it would be worthwhile for future studies to investigate more specific determinants of better and worse outcomes (e.g., certain mental health problems or family circumstances). This may help policy makers adjust their approach accordingly.

### Strengths and limitations

Our study has several strengths. First, by assessing mental health in three large samples at multiple, independent, cross‐sectional measurements before and since the pandemic, we avoided contamination of pandemic effects with treatment effects, developmental effects, and other time effects. For example, as children age, they are expected to report different mental health problems and as children go through treatment on average they will improve, but many longitudinal designs do not take such effects into account. Ideal designs include longitudinal measurements *within* cohorts that can be compared between *different* comparable cohorts, but such designs are often impossible because multiple pre‐covid measurements as well as multiple pandemic measurements must be available in similar but separate cohorts (see Burdzovic Andreas and Brunborg (2021) and Elmer et al. (2020) for good examples of such designs). Second, where most studies employ convenience samples, we included one long‐term cohort sample, one representative sample of the general population, and one sample of children receiving psychiatric care, reducing selection bias, and increasing generalizability to the whole spectrum of the child and adolescent population. Finally, we used well‐validated measures to assess mental health problems whereas a recent review concluded that fewer than 15% of available studies used validated instruments (Samji et al., 2022).

Our study also has several limitations. First, we mentioned the use of independent assessments as a strength of the study, but it also comes with the limitation that groups of participants differ for each measurement. Such differences for which we cannot control (e.g., genetic or shared environmental differences) may also impact child mental health. To test this, we performed a supplementary mixed linear model analysis using the longitudinal data of the NTR. The results showed highly similar patterns as our main analysis and are reported in the Supporting Information. Second, because of the lack of pre‐covid measurements in the clinical sample, we cannot assess whether mental health problems acutely increased since the start of the pandemic as we observed in the general population, or that it took longer for the pandemic to impact the mental health of these children. Theoretically, it is also possible that mental health in the clinical sample improved at the onset of the pandemic and then returned to pre‐pandemic levels, but this seems an unlikely possibility. Third, because the parent‐report and self‐report measures in the general population were collected in different samples, we cannot assess whether differences are due to different characteristics (for example the difference in age) or due to different reporters, as in general correlations between raters is limited (Roy et al., 2010) and raters provide both bias and unique views (Bartels et al., 2007). Fourth, the KLIK sample may suffer from volunteer bias, the NTR sample includes relatively many participants from high SES families, and although the DREAMS sample is representative of the population in specialized child psychiatric care (Fischer et al., 2022), low SES families are less likely to find their way into specialized care. Also, for twins, levels of isolation may have been lower. However, in general twins are considered equal to singletons for cognitive, emotional, and behavioral traits (Johnson et al., 2002; Reiss et al., 2000). Fifth, limited response rates in the DREAMS and NTR samples may have led to selection bias and although we controlled for background characteristics, we cannot rule out this has impacted results. Finally, other large‐scale events such as the war in Ukraine may have also impacted child mental health and we could not control for such effects. Similarly, we did not take lockdown specific effects, for example, curfews or school closing, on children's mental health into account.

## CONCLUSION

Internalizing child mental health problems increased during the pandemic in the general population and in children in psychiatric care. In the general population, problems were most prevalent during the first year of the pandemic. According to children (but not to parents) these have improved toward April 2022. Problems remain substantially higher than pre‐pandemic levels. In the clinical population, problems increased throughout the pandemic and have not improved since. In fact, mental health problems in some domains, such as depression and sleep impairments, have worsened up until April 2022 when social restrictions were largely relaxed again. We found no evidence of sex effects and limited evidence suggesting younger children might recuperate more slowly. Children from the general population seem to be more resilient to negative mental health effects of the pandemic than children in psychiatric care, in whom we see more long‐term effects. We stress the importance of monitoring mental health in children throughout and beyond the pandemic to aid health care and policy.

## AUTHOR CONTRIBUTIONS

**Josjan Zijlmans**: Conceptualization, Data curation, Formal analysis, Funding acquisition, Investigation, Methodology, Project administration, Visualization, Writing – original draft, Writing – review & editing. **Jacintha M. Tieskens**: Conceptualization, Data curation, Formal analysis, Investigation, Methodology, Visualization, Writing – original draft, Writing – review & editing. **Hedy A. van Oers**: Conceptualization, Data curation, Formal analysis, Investigation, Methodology, Writing – review & editing. **Hekmat Alrouh**: Conceptualization, Data curation, Formal analysis, Investigation, Methodology, Writing – review & editing. **Michiel A. J. Luijten**: Conceptualization, Data curation, Formal analysis, Investigation, Methodology, Writing – review & editing. **Rowdy de Groot**: Writing – review & editing. **Daniël vander Doelen**: Data curation, Writing – review & editing. **Helen Klip**: Data curation, Writing – review & editing. **Rikkert M. van der Lans**: Data curation, Writing – review & editing. **Ronald de Meyer**: Writing – review & editing. **Malindi van der Mheen**: Writing – review & editing. **Hyun Ruisch**: Data curation, Writing – review & editing. **Germie van den Berg**: Writing – review & editing. **Hilgo Bruining**: Writing – review & editing. **Jan Buitelaar**: Writing – review & editing. **Rachel van der Rijken**: Writing – review & editing. **Pieter J. Hoekstra**: Conceptualization, Funding acquisition, Writing – review & editing. **Marloes Kleinjan**: Writing – review & editing. **Ramón J. L. Lindauer**: Conceptualization, Funding acquisition, Writing – review & editing. **Kim J. Oostrom**: Writing – review & editing. **Wouter Staal**: Conceptualization, Funding acquisition, Writing – review & editing. **Robert Vermeiren**: Conceptualization, Funding acquisition, Writing – review & editing. **Ronald Cornet**: Writing – review & editing. **Lotte Haverman**: Conceptualization, Funding acquisition, Project administration, Supervision, Writing – review & editing. **Arne Popma**: Conceptualization, Funding acquisition, Project administration, Supervision, Writing – review & editing. **Meike Bartels**: Conceptualization, Funding acquisition, Project administration, Supervision, Writing – review & editing. **Tinca J. C. Polderman**: Conceptualization, Funding acquisition, Project administration, Supervision, Writing – review & editing.

## CONFLICTS OF INTEREST STATEMENT

MB is supported by a European Research Council consolidator Grant (WELL‐BEING 771057 PI Bartels). The authors have declared that they have no competing or potential conflicts of interest.

## ETHICAL CONSIDERATIONS

Collaborating parties received approval for data collection by the appropriate ethics committees and all children and parents provided informed consent. The studies were conducted in line with the ethical standards stated in the 1964 Declaration of Helsinki and its later amendments.

## Supporting information

Supporting Information S1

## Data Availability

Data available on request from the authors.
