# Supplementary material for: The effects of COVID‐19 on child mental health: Biannual assessments up to April 2022 in a clinical and two general population samples
Source: JCPP Adv. 2023 Mar 30;3(2):e12150. doi: 10.1002/jcv2.12150 (PMC10519731; doi:10.1002/jcv2.12150)
Supplement: Supplementary file 1 — Supporting Information S1 [file JCV2-3-e12150-s001.docx]

**Supporting Information**

*Table S1. BPM parent-report sum score estimated marginal means (EMM), standard errors, comparisons between measurement points, and % elevated scores*

| Cohort |  | 0 (a)  pre-pandemic | 1 (b)  Apr/May 2020 | 2 (c)  Nov/Dec 2020 | 3 (d)  Mar/Apr 2021 | 4 (e)  Nov/Dec 2021 | 5 (f)  Mar/Apr 2022 |
| --- | --- | --- | --- | --- | --- | --- | --- |
| NTR | N | 13341 | 1332 | 221 | 347 | 426 | 458 |
|  | BPM Internalizing | 0.89 (0.01)^bcdef^ | 1.45 (0.04)^adef^ | 1.24 (0.11)^adef^ | 1.77 (0.09)^abc^ | 1.71 (0.08)^abc^ | 1.72 (0.08)^abc^ |
|  | BPM Externalizing | 2.05 (0.02) | 2.07 (0.06) | 2.04 (0.14) | 2.25 (0.11) | 2.15 (0.10) | 2.11 (0.10) |
|  | BPM Int % elevated | 6.9% | 16.4% | 13.5% | 19.9% | 20.2% | 19.9% |
|  | BPM Ext % elevated | 8.2% | 7.8% | 9.0% | 11.5% | 8.5% | 8.5% |
|  |  |  |  |  |  |  |  |
| DREAMS | N | - | 404 | 599 | 445 | 413 | 295 |
|  | BPM Internalizing | - | 5.00 (0.21)^def^ | 5.18 (0.19)^de^ | 5.67 (0.20)^bc^ | 5.67 (0.20)^bc^ | 5.66 (0.23)^c^ |
|  | BPM Externalizing | - | 4.78 (0.21) | 4.99 (0.19) | 5.16 (0.20) | 5.05 (0.20) | 5.10 (0.23) |
|  | BPM Int % elevated | - | 62.9% | 64.6% | 72.8% | 72.2% | 71.5% |
|  | BPM Ext % elevated | - | 38.1% | 38.9% | 39.8% | 39.7% | 40.7% |

Note. ^a,b,c,d,e,f^ represent significant differences at *p* < .05 between measurements using Least Significant Differences post-hoc tests. E.g., superscript ^b^ in column (d) indicates a significant post-hoc difference between columns (b) and (d) for a variable.

*Table S2. PROMIS self-report T-score estimated marginal means (EMM), standard errors, and comparisons between measurement points*

| Cohort |  | 0 (a)  pre-pandemic | 1 (b)  Apr/May 2020 | 2 (c)  Nov/Dec 2020 | 3 (d)  Mar/Apr 2021 | 4 (e)  Nov/Dec 2021 | 5 (f)  Mar/Apr 2022 |
| --- | --- | --- | --- | --- | --- | --- | --- |
| KLIK | N | 527-1082* | 471-486 | 425-440 | 407-413 | 401-414 | 514-529 |
|  | Anxiety† | 43.6 (0.3)^bcdef^ | 50.4 (0.4)^af^ | 50.0 (0.5)^a^ | 50.1 (0.5)^a^ | 50.1 (0.5)^a^ | 49.1 (0.4)^ab^ |
|  | Depressive symptoms† | 44.7 (0.3)^bcdef^ | 49.3 (0.5)^a^ | 49.0 (0.5)^a^ | 49.9 (0.5)^af^ | 49.5 (0.5)^af^ | 48.2 (0.5)^ade^ |
|  | Sleep-related impairments† | 47.1 (0.5)^bcdef^ | 50.1 (0.6)^a^ | 50.6 (0.7)^a^ | 50.7 (0.6)^a^ | 50.7 (0.6)^a^ | 50.0 (0.5)^a^ |
|  | Anger† | 44.1 (0.5)^bcdef^ | 47.2 (0.6)^af^ | 47.3 (0.7)^af^ | 47.3 (0.6)^af^ | 47.6 (0.6)^af^ | 45.7 (0.5)^abcde^ |
|  | Global health‡ | 46.4 (0.4)^de^ | 45.6 (0.5)^d^ | 45.3 (0.6) | 44.2 (0.5)^abf^ | 45.2 (0.5)^a^ | 45.7 (0.5)^d^ |
|  | Peer relations‡ | 47.1 (0.4)^bcde^ | 44.2 (0.5)^acdef^ | 45.7 (0.6)^ab^ | 45.6 (0.5)^abf^ | 45.8 (0.5)^ab^ | 46.8 (0.5)^bd^ |
|  |  |  |  |  |  |  |  |
| DREAMS | N | - | 241-257 | 434-456 | 236-250 | 199-216 | 169-181 |
|  | Anxiety† | - | 51.9 (0.7)^cdef^ | 54.1 (0.7)^b^ | 53.8 (0.7)^be^ | 55.6 (0.7)^bd^ | 54.3 (0.8)^b^ |
|  | Depressive symptoms† | - | 52.9 (0.8)^cdef^ | 55.1 (0.7)^b^ | 55.8 (0.8)^b^ | 55.4 (0.8)^b^ | 56.5 (0.9)^b^ |
|  | Sleep-related impairments† | - | 54.0 (0.7)^cdef^ | 55.6 (0.7)^b^ | 55.8 (0.8)^b^ | 55.9 (0.8)^b^ | 56.7 (0.9)^b^ |
|  | Anger† | - | 51.6 (0.8)^cdef^ | 53.6 (0.7)^b^ | 54.2 (0.8)^b^ | 53.8 (0.8)^b^ | 54.0 (0.9)^b^ |
|  | Global health‡ | - | 42.1 (0.6)^def^ | 41.0 (0.6)^d^ | 39.0 (0.6)^bc^ | 40.0 (0.7)^b^ | 39.5 (0.7)^b^ |
|  | Peer relations‡ | - | 43.2 (0.7) | 43.9 (0.6)^de^ | 41.8 (0.7)^c^ | 43.0 (0.7) | 42.1 (0.8)^c^ |

Note. ^a,b,c,d,e,f^ represent significant differences at *p* < .05 between measurements using Least Significant Differences post-hoc tests. E.g., superscript ^b^ in column (d) indicates a significant post-hoc difference between columns (b) and (d) for a variable.

* Sample sizes vary because data from different domains comes from different norm studies.

† Higher scores indicate more symptoms

‡ Higher scores indicate better functioning

*Table S3. PROMIS % normal, moderately elevated, and severely elevated scores*

* Sample sizes vary because data from different domains comes from different norm studies.

|  |  |  | KLIK |  |  |  |  |  | DREAMS |  |  |  |  |  |
| --- | --- | --- | --- | --- | --- | --- | --- | --- | --- | --- | --- | --- | --- | --- |
|  |  |  | 0 (a)  pre-pandemic | 1 (b)  Apr/May 2020 | 2 (c)  Nov/Dec 2020 | 3 (d)  Mar/Apr 2021 | 4 (e)  Nov/Dec 2021 | 5 (f)  Mar/Apr 2022 | 0 (a)  pre-pandemic | 1 (b)  Apr/May 2020 | 2 (c)  Nov/Dec 2020 | 3 (d)  Mar/Apr 2021 | 4 (e)  Nov/Dec 2021 | 5 (f)  Mar/Apr 2022 |
| N |  |  | 527-1082* | 471-486 | 425-440 | 407-413 | 401-414 | 514-529 | - | 241-257 | 434-456 | 236-250 | 199-216 | 169-181 |
| Anxiety |  | normal | 74.8% | 46.6% | 50.9% | 51.0% | 52.9% | 56.4% | - | 45.2% | 32.7% | 35.7% | 29.7% | 36.9% |
|  |  | moderate | 20.0% | 49.3% | 43.1% | 39.0% | 37.3% | 35.4% | - | 40.8% | 43.3% | 49.2% | 45.8% | 42.6% |
|  |  | severe | 5.2% | 4.2% | 6.0% | 10.0% | 9.8% | 8.2% | - | 14.0% | 24.0% | 15.2% | 24.5% | 20.5% |
| Depressive |  | normal | 74.8% | 60.0% | 61.2% | 55.5% | 60.6% | 63.8% | - | 52.2% | 38.6% | 38.8% | 39.5% | 34.1% |
| Symptoms |  | moderate | 20.1% | 37.2% | 33.2% | 37.9% | 32.2% | 31.0% | - | 31.0% | 32.9% | 37.6% | 41.0% | 38.2% |
|  |  | severe | 5.1% | 2.7% | 5.6% | 6.6% | 7.2% | 5.2% | - | 16.7% | 28.5% | 23.6% | 19.5% | 27.7% |
| Sleep-related |  | normal | 71.5% | 64.5% | 58.2% | 55.4% | 55.9% | 61.8% | - | 50.2% | 39.2% | 39.8% | 39.8% | 36.0% |
| impairments |  | moderate | 23.3% | 33.4% | 37.3% | 40.0% | 39.4% | 34.9% | - | 38.7% | 44.7% | 47.0% | 46.3% | 43.6% |
|  |  | severe | 5.1% | 2.1% | 4.5% | 4.7% | 4.7% | 3.3% | - | 11.1% | 16.1% | 13.1% | 13.9% | 20.3% |
| Anger |  | normal | 75.0% | 73.5% | 69.6% | 68.5% | 68.0% | 75.9% | - | 54.9% | 46.7% | 41.5% | 45.9% | 45.1% |
|  |  | moderate | 19.9% | 23.6% | 25.3% | 24.6% | 24.9% | 20.7% | - | 28.9% | 35.1% | 42.3% | 37.3% | 39.4% |
|  |  | severe | 5.1% | 2.9% | 5.1% | 6.8% | 7.1% | 3.4% | - | 16.3% | 18.1% | 16.2% | 16.7% | 15.4% |
| Global |  | normal | 75.0% | 72.0% | 66.8% | 66.3% | 68.8% | 71.3% | - | 59.9% | 44.3% | 41.2% | 46.8% | 39.2% |
| Health |  | moderate | 20.1% | 26.3% | 27.7% | 27.8% | 25.4% | 23.4% | - | 27.2% | 29.6% | 34.0% | 31.0% | 31.5% |
|  |  | severe | 5.0% | 1.6% | 5.5% | 5.8% | 5.8% | 5.3% | - | 12.8% | 26.1% | 24.8% | 22.2% | 29.3% |
| Peer |  | normal | 74.2% | 71.3% | 77.2% | 75.4% | 76.8% | 79.6% | - | 66.0% | 68.7% | 55.5% | 63.3% | 60.9% |
| relations |  | moderate | 19.9% | 22.9% | 17.4% | 19.7% | 17.7% | 14.8% | - | 22.4% | 23.0% | 34.3% | 28.6% | 29.0% |
|  |  | severe | 5.9% | 5.7% | 5.4% | 4.9% | 5.5% | 5.6% |  | 11.6% | 8.3% | 10.2% | 8.0% | 10.1% |

*Table S4. Supplementary analysis with pre-pandemic data up to five years before the pandemic: BPM parent-report sum score estimated marginal means (EMM), standard errors, comparisons between measurement points*

| Cohort |  | 0 (a)  pre-pandemic | 1 (b)  Apr/May 2020 | 2 (c)  Nov/Dec 2020 | 3 (d)  Mar/Apr 2021 | 4 (e)  Nov/Dec 2021 | 5 (f)  Mar/Apr 2022 |
| --- | --- | --- | --- | --- | --- | --- | --- |
| NTR | N | 1214 | 1332 | 221 | 347 | 426 | 458 |
|  | BPM Internalizing | 0.78 (0.06)^bcdef^ | 1.41 (0.06)^adef^ | 1.28 (0.13)^adef^ | 1.84 (0.11)^abc^ | 1.73 (0.10)^abc^ | 1.72 (0.09)^abc^ |
|  | BPM Externalizing | 1.77 (0.07)^bcdef^ | 2.11 (0.07)^a^ | 2.15 (0.15)^a^ | 2.39 (0.13)^a^ | 2.24 (0.11)^a^ | 2.16 (0.11)^a^ |

Note. ^a,b,c,d,e,f^ represent significant differences at *p* < .05 between measurements using Least Significant Differences post-hoc tests. E.g., superscript ^b^ in column (d) indicates a significant post-hoc difference between columns (b) and (d) for a variable.

**Supplementary analyses**

For the NTR general population, within-person data exists for all measurement moments, including the pre-covid measurements. Therefore, we modeled a pre-pandemic versus during covid variable, a variable representing all COVID measurements, and a variable representing the quadratic effect of all COVID measurements. In addition, we included age, sex, and interactions between time-variables and age and sex.

We carried out the linear mixed model analysis using nlme package (version 3.1-159) in R (version 4.1.0). We estimated the model using a maximum likelihood estimator. The following equation describes the model: Y_ij_ = (β_1_ + b_1i_ ) + (β_2_ + b_2i_ ) * Cov  + (β_3_ + b_3i_ ) * time + (β_4_ +b_4i_) * time^2^ + β_5_ * sex + β_6_ * age + β_7_* Cov * sex + β_8_* Cov * age + β_9_* time * sex + β_10_* time * age + e_ij_,  where Y_ij_ is the internalizing/externalizing score for individual i at measurement j , Cov is the variable specifying a measurement during the pandemic, e_ij_ is the error term, β1 , …, β10 are the fixed effects (population averages) and b_1i_, …, b_4i_ are the individual specific random intercept and slopes.

We observed a significant increase in internalizing scores from pre-pandemic to pandemic measurements (t = 3.46, p < .001), with no significant changes in subsequent measurements. There was a significant interaction between pre-pandemic versus pandemic and age (t = -2.09, p < .05) with younger children showing larger increases in internalizing problems, but no significant interaction with sex. There was a significant interaction between time during COVID and sex (t = 1.98, p < .05), with girls having higher internalizing problems over time, but no significant interaction with age.

We observed a significant increase in externalizing scores from pre-pandemic to pandemic measurements (t = -2.86, p < .01), with no significant changes in subsequent measurements. There were no significant interactions between start of covid lockdown measures and age or sex, nor between time during covid and age, but there was a significant interaction between time during covid and sex (t = 2.58, p < 05).
